# Retrospective Analysis of Characteristics, Indications and Outcomes of Endoscopic Retrograde Cholangiopancreatography (ERCP) in a Tertiary Referral Center in Saudi Arabia

**DOI:** 10.7759/cureus.36794

**Published:** 2023-03-28

**Authors:** Laeeque A Qureshi, Adnan Alzanbagi, Abdulaziz Tashkandi, Mohammed S Khan, Zaffar M Malik, Mohammed E Hefny, Mohammed K Shariff

**Affiliations:** 1 Digestive and Liver Center (DLC) and Advanced Endoscopy Center, King Abdullah Medical City, Makkah, SAU; 2 DLC & Advanced Endoscopy Center, King Abdullah Medical City, Makkah, SAU

**Keywords:** treatment of cholangiocarcinoma, jaundice, perforation, stent, pancreatitis, bleeding, cbd, ercp, ercp cbd complication bleeding pancreatitis stent

## Abstract

Background: Endoscopic retrograde cholangiopancreatography (ERCP) is an advanced endoscopic procedure that is an essential tool in the management of pancreaticobiliary diseases. There is limited data available on the indications and outcomes of ERCP from this region. Therefore, we aim to report the characteristics of patients, indications and outcomes of ERCP in Saudi Arabia.

Methods: We retrospectively looked at ERCP procedures done at a tertiary referral center covering the western region of Saudi Arabia from August 2018 until July 2020. Data were collected from the hospital's electronic patient record and endoscopy database.

Results: Of 1001 ERCPs performed, full data was available on 712 procedures on 581 patients that were included in the final analysis. Mean age was 53.1 years. Four hundred four (56.7%) were female. Board-certified consultants performed all ERCPs. The most common intervention was sphincterotomy, which was performed in 563 (96.9%) patients who underwent first-ever ERCP, followed by dilatation and stenting. The commonest indication of ERCP was confirmed or suspected choledocholithiasis (52.6%), followed by replacement or removal of a biliary stent (15.7%), 55 (7.7%) for suspected ascending cholangitis, 54 (7.5%) for acute biliary pancreatitis and 15 (2%) for suspected sphincter of Oddi dysfunction. The commonest finding among all patients was choledocholithiasis in 57.9%, debris in 15.2% and biliary stricture in 14.8%. The commonest complication was pancreatitis in 22 (3.1%) followed by post-sphincterotomy bleeding in 16 (2.2%) and perforation in nine (1.2%). Bleeding was controlled by endoscopic intervention in four (25%) and one (6.2%) patient underwent surgery. One (0.14%) patient had procedure-related mortality secondary to post-procedure pulmonary embolism and one had significant morbidity and prolonged hospitalization because of complicated perforation. The deeper common bile duct (CBD) cannulation rate was 97.3%.

Conclusion: Our study results revealed that ERCP performed in the western region of Saudi Arabia has similar indications and findings as reported in the international literature. ERCP is successful in achieving the therapeutic objectives with complication rates consistent with published data.

## Introduction

Endoscopic retrograde cholangiopancreatography (ERCP) is an advanced endoscopic procedure that has been an essential tool in the diagnosis and treatment of biliary and pancreatic diseases since its introduction in 1968 [1]. In the past, ERCP was only for diagnostic purposes, but now it is almost exclusively a therapeutic tool. About 350,000-500,000 ERCPs are performed annually in the United States [2]. ERCP is currently the procedure of choice for many pancreaticobiliary diseases. There are multiple indications for ERCP including choledocholithiasis, biliary stricture, primary sclerosing cholangitis, biliary leak, and sphincter of Oddi dysfunction [3]. In addition, ERCP is indicated as a palliative treatment for obstructive jaundice caused by cholangiocarcinoma, or pancreatic cancer [3-4]. ERCP has considerable risks including severe complications and even death. The most common complication of ERCP is pancreatitis, which can happen due to several factors [5-6]. Other complications include cholangitis, cardiopulmonary events mainly due to sedation, bleeding and perforation [5-6]. Overall, the reported post-ERCP morbidity rate is 6.9% and a mortality of 0.3% in the adult population [7]. Globally, several studies were conducted to assess the indications and outcomes of ERCP [8-9]. Apart from a single retrospective study reporting on small numbers, there is limited data about different indications and outcomes of ERCP in Saudi Arabia [10].

Therefore, the aim of our study is to report the characteristics of patients who underwent ERCP at a single tertiary referral center in the Kingdom of Saudi Arabia (KSA). 

## Materials and methods

Research setting

King Abdullah Medical City (KAMC) is a tertiary healthcare facility in Makkah city, Kingdom of Saudi Arabia (KSA). Digestive and Liver Center (DLC) & Advanced Endoscopy Center (AEC) is one of the busiest units of KAMC, receiving patient from the entire western region of KSA. Approximately 500 ERCP procedures are performed each year.

Design and study population

This was a retrospective cross-sectional study that included all patients above the age of 18 years who had ERCP performed in DLC & AEC from 2018 to 2020.

Study outcomes

The primary outcome was to evaluate the common indications and outcomes of patients who underwent ERCP at KAMC. The secondary outcome was to report procedure-related complications including pancreatitis, bleeding, perforation, and cardiopulmonary complications.

Study procedure

Ethical approval was obtained from King Abdullah Medical City, Makkah institutional review board that has been accredited by the Association for the Accreditation of Human Research Protection Program. The hospital medical e-record system (Track Care, InterSystems) and endoscopy reporting system (Endobase) were searched for all ERCP done during the period of January 2018 to February 2020. Data was collected on (1) Patient demographics (2) ERCP indications (3) Procedural characteristics and findings and (4) All possible intra-procedural and post-procedural complications. 

Indications of ERCP were assessed in patients with obstructive jaundice confirmed by pre-procedure investigations including blood work and radiology either ultrasonography (US), computed tomography (CT) scan, or magnetic resonant cholangiopancreatography (MRCP). Written consent was obtained from every patient explaining procedure details, risks, and benefits and all patients were evaluated by anesthesia for sedation or general anesthesia for the procedure. 

All ERCP procedures were performed by five board-certified endoscopists who had completed a minimum of 500 bile duct cannulations. The procedures were performed in a dedicated fluoroscopy unit located in the endoscopy department with all sedation provided by a physician anesthesiologist.

## Results

The details of the procedure were documented in the hospital’s database (Endobase) by the endoscopist who performed the ERCP. The retrospective analysis of 712 ERCP was made according to the objectives of the study. The baseline characteristics and indications are shown in Table 1.

**Table 1:** Characteristics and indications of endoscopic retrograde cholangiopancreatography (ERCP) (n=712)

Characteristics	Number of Procedures (%)
Age __mean (SD):	53.10 (18.93)
Gender:	
Women	404 (56.7%)
Men	308 (43.3%)
Primary indications for ERCP:	
Suspected or confirmed stones	375 (52.6%)
Replacement or removal of biliary stent	81 (11.3%)
Cholangitis	55 (7.7%)
Acute biliary pancreatitis	54 (7.58%)
Suspected or confirmed stricture	43 (6.03%)
Bile Leak	36 (5.05%)
Stent placement across strictures, fistula, post-operative bile leak, or large common bile duct stones.	22 (3.08%)
? Sphincter of Oddi Dysfunction (SOD)	15 (2%)

**Figure 1:**
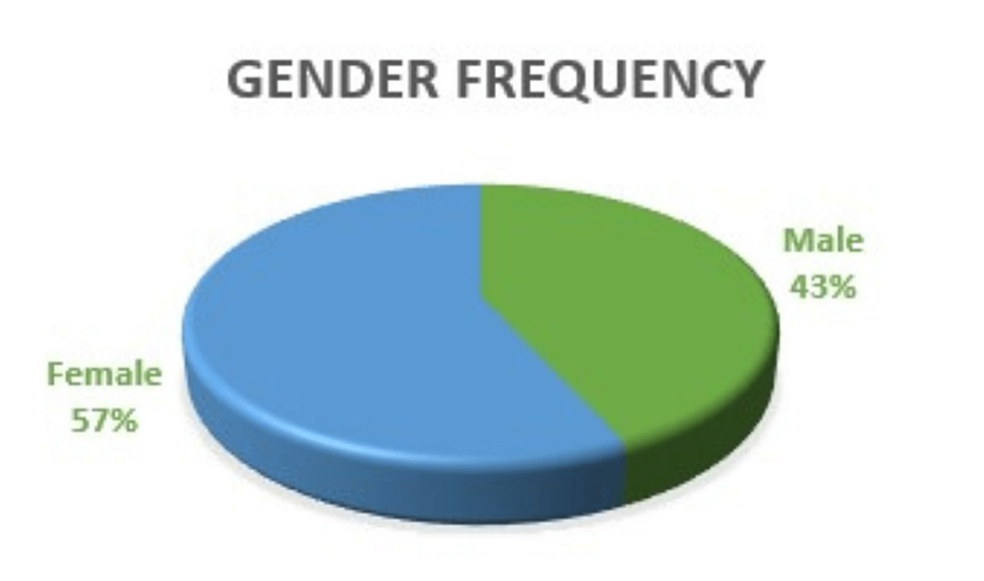
Gender distribution among patients who underwent endoscopic retrograde cholangiopancreatography (ERCP)

They had a mean age of 53 (±18) and 57% of them were women while 43% were men (Figure 1).

**Figure 2:**
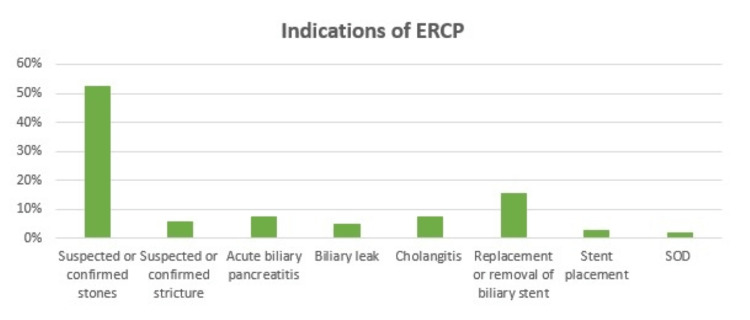
Indications of endoscopic retrograde cholangiopancreatography (ERCP) SOD: sphincter of Oddi dysfunction

Most patients had ERCP for suspected choledocholithiasis (52.6%) followed by replacement or removal of a biliary stent (15.7%), then cholangitis (7.7%), acute biliary pancreatitis (7.5%), suspected stricture (6.0%), biliary leak (5.0%), stent placement (3.0%), and the least was sphincter of Oddi dysfunction (2%) as shown in Figure 2. The common ERCP-related complications are shown in Table 2.

**Table 2:** Incidence of procedure-related complications

Characteristics	Number of Procedures (%)
Pancreatitis	16 (2.2%)
Bleeding	2 (0.3%)
Bleeding stooped by:	
Spontaneously	10 (62.5%)
Endoscopic Intervention	4 (25%)
Epinephrine Injection	1 (6.25%)
Surgery	1 (6.25%)
Termination of the procedure. Yes No	1 (6.25%) 15 (93.75%)
Patient needed blood transfusion:	4 (25%)
Patient needed admission to the ICU	4 (25%)
Perforation	2 (0.3%)
Death	1 (0.1%)

Bleeding from the sphincterotomy site was the most overserved ERCP complication; it accounted for 2.2% where 25% of them needed a blood transfusion and admission to the intensive care unit. It was followed by pancreatitis at 0.3% and perforation at 0.3%. Prolonged hospital stay due to a complicated perforation was recorded in one patient (0.1%). Death because of air embolism was reported in one patient (0.1%).

Details of procedural outcomes and characteristics of biliary system are shown in Table 3.

**Table 3:** Outcomes of endoscopic retrograde cholangiopancreatography (ERCP) and characteristics of biliary system. CBD: common bile duct; CHD: common hepatic duct; IHD: intrahepatic bile duct

Characteristics	Number of Procedures (%)
Procedural success rate	693 (97.3%)
Commonest finding among men & women:	
Stones (Women)	252 (61%)
Stones (Men)	160 (38.8%)
ERCP findings:	
Biliary Stones	375 (57.9%)
Strictures	108 (15.2%)
Debris or sludge	107 (15%)
Cystic duct stone	37 (8.9%)
Pus	30 (4.2%)
Bile Leak	26 (3.7%)
Fistula	9 (1.3%)
Worms	1 (0.1%)
Successful complete stones extraction	354 (93.9%)
Size of stones __mean (SD): (mm)	11.47 (11.9)
Lithotripsy	111 (15.6%)
Mechanical	103 (14.5%)
Laser	6 (0.8%)
Emergency crush	2 (0.3%)
Stent placement/ Replacement	191 (26.8%)
Type of stent:	
Plastic	159 (83.2%)
­­Metallic	32 (16.7%)
Size of stent __mean (SD): (mm)	80.91 (17.3)
Biliary system characteristics:	
Ampulla status	
Normal	509 (71.5%)
With biliary stent	81 (11.3%)
Fissured	31 (4.4%)
Inside or at the edge of diverticulum	21 (2.9%)
With Precut	20 (2.8%)
Growth	16 (2.2%)
Floppy	7 (1%)
Ulcerated	5 (0.7%)
Congested	2 (0.3%)
Swollen	1 (0.1%)
Not Seen or reached	19 (2.7%)
CBD status:	
Dilated	412 (57.8%)
Normal	297 (41.7%)
Tortuous	3 (0.4%)
CHD status:	
Normal	515 (72.3%)
Dilated	101 (14.1%)
Not seen	77 (10.8%)
IHD status:	
Normal	531 (74.6%)
Dilated	101 (14.1%)
Beaded	8 (1 %)
Pancreatic duct status:	
Normal	81 (11.4%)
Dilated	19 (2.7%)

Outcomes include the findings on cholangiogram such as stones, strictures, or bile leak and successful biliary stone removal or stent placement etc. Success of procedure is considered as rate of deeper common bile duct (CBD) cannulation and was achieved in 97.3% of cases. The commonest finding among men and women is the biliary stone, either intra- or extrahepatic or inside cystic duct or gall bladder. It is followed by debris or sludges (15.2%), strictures (12.1%), pus (4.2%), biliary leak (3.2%), and fistula (1.2%). Complete stones extraction was successful in 354 (93.9%) cases. The characteristics of the biliary system include ampullary position and shape, size and shape of CBD, common hepatic duct (CHD), intra-hepatic duct (IHD), and cystic ducts and pancreatic duct in selected cases.

## Discussion

ERCP has shown and emphasized its importance as a therapeutic tool for many pancreaticobiliary diseases including common problems such as choledocholithiasis [3]. To accurately determine how much ERCP is an appropriate tool, it’s important to report and review the most updated data regarding the possible complications and outcomes. The present study is evaluating the different aspects of ERCP, i.e. common indications and outcomes and biliary characteristics of patients who underwent ERCP in our center.

As shown by different reports around the world, suspected or confirmed stones is the most frequent indication for ERCP [10-12]. The latest study was published in 2020 and it was at a Canadian tertiary center that showed 61% of patients who had ERCP is for suspected or confirmed stones followed by suspected or confirmed strictures [10], which is the same in this region too. Similarly, the rate of complications is comparable to the reported results in the same study. By looking at Figure 2, choledocholithiasis is still the most frequently reported indication, followed by replacement or removal of a biliary stent as the most probable cause of obstruction. In another report from Finland, palliative ERCP for pancreaticobiliary malignancies is the second commonest indication [11], also noted same in our study.

Determining precisely the procedure’s risk factors and implementing the appropriate measures such as pharmacological measures to reduce the rate of ERCP-related pancreatitis is an important step to prevent ERCP-related complications which eventually is going to impact the outcomes in a positive way [12]. According to multiple reports, pancreatitis is the most commonly observed complication followed by bleeding and perforation [12]. Cardiopulmonary complications and death are the lowest observed complications [12]. In the present study, pancreatitis is the second reported ERCP-related complication where intra-operative bleeding is the commonest seen complication. With the surgical risks and adverse events especially among the elderly and those who have co-morbidities, ERCP has been showing its safety and efficacy with a relatively low rate of complications [13]. Cardiopulmonary complications and death are the lowest reported and the overall ERCP-related mortality rate is 0.1%. It was a result of air embolism, which is potentially life-threatening but also, it’s a rare complication. 

By comparing ERCP findings among men and women, it appears that women are more at risk of having choledocholithiasis than men. This explains the higher number of female patients undergoing ERCP.

There are some limitations to the study where further details such as BMI, type of sedative or non-steroidal anti-inflammatory drugs (NSAIDs) that were used, and cannulation time haven’t been reported because they were missing and not documented for some patients.

## Conclusions

ERCP is a useful therapeutic procedure that has shown promise in treating a variety of pancreaticobiliary disorders, particularly those with significant surgical risk. As a result of the procedure's invasiveness, problems can arise. Prior to the treatment, patients with advanced comorbid conditions should have a thorough evaluation. Data from our center is almost similar as reported from other centers. An advanced endoscopist, anesthesiologists, gastrointestinal nurses, and a hepatobiliary surgeon can all work together as a multidisciplinary team to improve the procedure's safety, outcomes, and success.
